# The Adirondacks as a Health Resort

**Published:** 1886-04

**Authors:** 


**The Adirondacks as a Health Resort,** showing the benefit to be derived by a sojourn in the wilderness, in cases of Pulmonary Phthisis, Acute and Chronic Bronchitis, Asthma, "Hay Fever" and various nervous affections. Edited by JOSEPH W. STICKLER, M. S., M. D. Published by G. P. Putnam's Sons. 1886.

This is an attractive little volume of some 200 pages, written with a design to show the advantages of the Adirondack region in the treatment of many affections known to be benefitted by change of climate. Physicians have been too ready to send their patients on long tedious journeys to distant states or countries. If the advantages claimed by Dr. Stickler for the Adirondack region are a fact, as he seems to demonstrate, we can often obtain equal advantages here near our homes without the inconvenience or expense of a long journey, and if no good results are obtained, it is no difficult matter to return home. The book is largely made up of letters from persons who have experienced the benefits derived from this mountain air, among others Drs. A. L. Loomis and J. R. Levick.

